# Robotic radical nephrectomy for renal cell carcinoma: a systematic review

**DOI:** 10.1186/1471-2490-14-75

**Published:** 2014-09-18

**Authors:** Anastasios D Asimakopoulos, Roberto Miano, Filippo Annino, Salvatore Micali, Enrico Spera, Beniamino Iorio, Giuseppe Vespasiani, Richard Gaston

**Affiliations:** 1 UOC of Urology, Department of Experimental Medicine and Surgery, University of Tor Vergata, Policlinico Casilino, Rome, Italy; 2 UOC of Urology, Department of Experimental Medicine and Surgery, University of Tor Vergata, Policlinico Tor Vergata Foundation, Rome, Italy; 3 U.O. Urologia, Ospedale San Donato, Arezzo, Italy; 4 Division of Urology, Clinique Saint-Augustin, Bordeaux, France; 5 Department of Urology, University of Modena and Reggio Emilia, Modena, Italy

**Keywords:** Robotic nephrectomy, Robotic radical nephrectomy, Robot-assisted laparoscopic radical nephrectomy, Radical nephrectomy, Renal cell carcinoma

## Abstract

**Background:**

Laparoscopic radical nephrectomy (LRN) is the actual gold-standard for the treatment of clinically localized renal cell carcinoma (RCC) (cT1-2 with no indications for nephron-sparing surgery). Limited evidence is currently available on the role of robotics in the field of radical nephrectomy. The aim of the current study was to provide a systematic review of the current evidence on the role of robotic radical nephrectomy (RRN) and to analyze the comparative studies between RRN and open nephrectomy (ON)/LRN.

**Methods:**

A Medline search was performed between 2000–2013 with the terms “robotic radical nephrectomy”, “robot-assisted laparoscopic nephrectomy”, “radical nephrectomy”. Six RRN case-series and four comparative studies between RRN and (ON)/pure or hand-assisted LRN were identified.

**Results:**

Current literature produces a low level of evidence for RRN in the treatment of RCC, with only one prospective study available. Mean operative time (OT) ranges between 127.8-345 min, mean estimated blood loss (EBL) ranges between 100–273.6 ml, and mean hospital stay (HS) ranges between 1.2-4.3 days. The comparison between RRN and LRN showed no differences in the evaluated outcomes except for a longer OT for RRN as evidenced in two studies. Significantly higher direct costs and costs of the disposable instruments were also observed for RRN. The comparison between RRN and ON showed that ON is characterized by shorter OT but higher EBL, higher need of postoperative analgesics and longer HS.

**Conclusions:**

No advantage of robotics over standard laparoscopy for the treatment of clinically localized RCC was evidenced. Promising preliminary results on oncologic efficacy of RRN have been published on the T3a-b disease. Fields of wider application of robotics should be researched where indications for open surgery still persist.

## Background

Renal cell carcinoma (RCC) together with renal pelvis cancer represents the 3.8% of all new cancer cases in the US, with an estimated 63920 new cases and 13860 cancer–related deaths in 2014 [1]. Laparoscopic radical nephrectomy (LRN) is the actual gold-standard for the treatment of clinically localized RCC (cT1-2 with no indications for nephron-sparing surgery) [2].

The first robotic radical nephrectomy (RRN) for RCC was described by Klingler et al. [3] in 2000. Before that, only feasibility studies of simple robotic nephrectomy on the suine model [4], one robotic nephrectomy on human for a case of a nonfunctioning hydronephrotic kidney -owing to a ureteropelvic junction obstruction- [5] and 11 robotic live-donor nephrectomies [6,7] were published, with promising results.

Aim of the current manuscript was to perform a systematic review of the current evidence on the role of RRN. The available comparative studies between RRN and ON/LRN were also analyzed.

## Methods

We performed a MedLine search for peer-reviewed studies, published from January 2000- December 2013, on the RRN for RCC. The keywords used were “robotic radical nephrectomy”, “robot-assisted laparoscopic nephrectomy”, “radical nephrectomy”.

Two individuals independently screened the titles and abstracts of each citation. The reference lists of the eligible articles were reviewed and the “Related citations” PubMed feature was utilized. Comparative studies between RRN and LRN or ON were also included in this review.

Abstracts and manuscripts reporting <5 cases or referring on robotics for live-donor nephrectomy, nephroureterectomy, partial nephrectomy or on robotic simple nephrectomy were excluded. Manuscripts in languages other than English were not considered.

Manuscripts have been assessed according to their level of scientific evidence (Oxford Center for Evidence-based Medicine, March 2009) [8].

## Results

**Figure 1:**
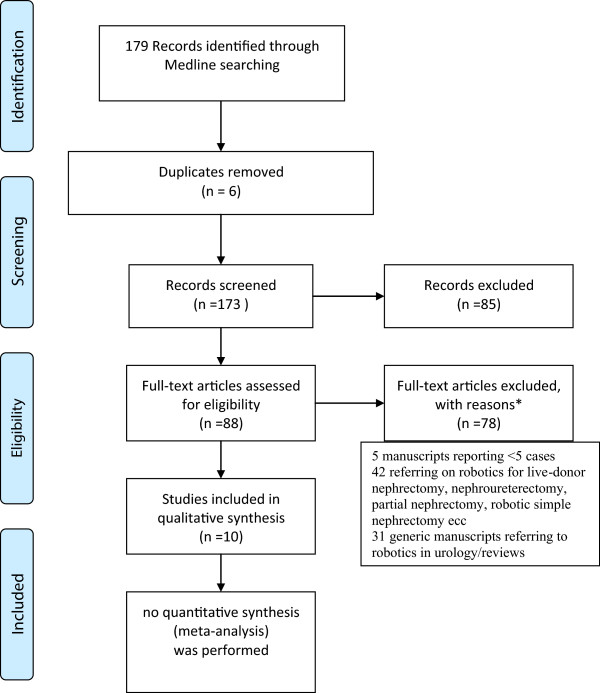
**Flow of information through the different phases of this systematic review, according to the PRISMA Flow Diagram (http://www.prisma-statement.org/statement.htm).**

One hundred and seventy-nine manuscripts were initially identified. Figure 1 provides a four-phase diagram on the flow of information through the different phases of this systematic review. By the application of the exclusion criteria described above, ten manuscripts were ultimately eligible for inclusion in this systematic review. Six studies were classified as case-series with a retrospective evaluation of the reported data [3,9-13], three studies were classified as case-comparative series with a retrospective evaluation of the reported data [14-16] while one study was a prospective case–control study [17]. Nine manuscripts refer on the traditional multi-port robotic access [3,9-13,15-17] while one on the single-site approach (robotic laparoendoscopic single-site surgery (R-LESS)) [14]. The transperitoneal route was mainly adopted. Several techniques of hilar control are described, such as endovascular stapler [3], hemoclips [13], Hemolock clips [17] or suture ligation/robotic Hemolock clips/staplers [9], based on the preferences of the operating surgeons.

Tables 1 and 2 refer on the perioperative and oncologic data of the published series, respectively. Table 3 reports on the described complications.

**Table 1:** Perioperative outcomes of RRN for RCC

**Author**	**Pts**	**BMI**	**OT (min)**	**EBL (ml)**	**Morph. Eq (mg)**	**HS (d)**	**Tumor size**	**Conversion**	**Level of evidence**
Klingler [3]	5	28	321 (M)	150 (M)	28 (M)	3 (M)	66 cm^3^ (M)	1 → HA-LRN	IV
Rogers [9]	35	30.5	291 (m)	221 (m)	18.5 (m)	2.5 (m)	5.1 cm (m)	None	IV
Dogra [10]	23	NR	132.7 (m)	270 (m)	NR	3 (m)	6.38 cm (m)	3 → ON	IV
Rogers [11]	18	NR	224 with 4th arm, 322 without (m)	NR	NR	NR	NR	NR	IV
Abaza [12]	5	36.6	327(m)	170 (m)	NR	1.2 (m)	10.4 cm (m)	None	IV
Nazemi [15]	6	27.6	345 (M)	125 (M)	19 (M)	3 (M)	4.5 cm (M)	1 → HA-LRN	IV
Hemal [17]	15	28.3	221 (m)	210 (m)	14.3 (m)	3.5 (m)	6.7 cm (m)	1 → ON	IIIb
Boger [16]	13	29	168 (M)	100 (m)	30 (m)	2 (m)	4.8 cm (m)	1 → LRN	IV
Lorenzo [13]	38	24.3	127.8 (m)	273.6 (m)	NR	4.3 (m)	NR	None	IV
White [14]	10	28.7	167.5 (M)	100 (M)	25.3 (M)	2.5 (M)	4.8 cm (M)	None	IV

Pts = patients, BMI = body mass index, OT = operative time, EBL = estimated blood loss, Morph.Eq = morphine equivalents, HS = hospital stay. Level of evidence IV = case-series (and poor quality cohort and case–control studies), IIIb = individual case–control study. NR = not reported, HA-LRN = hand-assisted laparoscopic radical nephrectomy, ON = open nephrectomy. m = mean, M = median.

**Table 2:** Oncologic outcomes of RRN for RCC

**Author**	**Mean (m) or median (M) follow-up (mo)**	**PSM**	**Local or distant recurrence**
Rogers [9]	15.7 (m)	0	No
Dogra [10]	29.4 (m)	0	No
Nazemi [15]	4 (M)	0	No
Hemal [17]	8.3 (m)	NR	No
Abaza [12]	15.4 (m)	0	No
Klingler [3]	NR	0	NR
Lorenzo [13]	12 (M)	0	No
White [14]	10.5 (m)	NR	NR
Boger [16]	NR	NR	NR
Rogers [11]	NR	NR	NR

NR = not reported.

**Table 3:** Complications/conversions of RRN for RCC (where reported)

Rogers [11]	4 wound dehiscences in morbidly obese patients
Nazemi [15]	1 stapler failure: renal vein bleeding and conversion in HA-LRN
Hemal [17]	2 vascular complications, 1 wound infection, 2 ileus
Boger [16]	2 pulmonary embolism, 1 pancreas injury, 1 liver laceration
Dogra [10]	Hilar bleeding with necessity of transfusion, 1 transfusion, 2 fever, 1 vomiting, 1 wound infection, 1 atrial fibrillation
Lorenzo [13]	7.9% transfusion rate
White [14]	Skin infection
Abaza [12]	None reported
Klingler [3]	1 bleeding requiring conversion to HA-LRN

## Discussion

While the first RRN was described in 2000 [3] only few studies have been published until now; the utility of the robotic platform in the field of RN, in fact, has been questioned mainly due to concerns such as higher costs, set up time, absence of force feedback and longer global operative times compared to LRN. Furthermore, in case of conversion for significant vascular complications, the initial part of the surgery has to be managed by the bed-side assistant, before the main surgeon scrubs in [10,11].

Overall, the current literature provides a low level of evidence for RRN in the treatment of RCC. The majority of the studies are small retrospective case-series with limited follow-up; only one case–control study reports a prospective evaluation of the collected data [17].

The transperitoneal route was the preferable way for accessing the kidney fossa. However, robotic assistance may also facilitate the retroperitoneal approach, offering precise dissection in a confined working space. Rogers et al. [9] describe the retroperitoneal RRN for three patients, two with extensive prior abdominal surgery and one on peritoneal dialysis.

As shown in Table 1, RRN is performed in acceptable operative times, with low EBL and conversion rates. The highest number of conversions to ON is reported in the study of Dogra et al. [10]; the primary reason for conversion was bleeding in all these cases.

RRN also provides acceptable, short-term oncologic efficacy (Table 2), both for the clinically localized and locally advanced RCC, even in cases of RCC with thrombus of the inferior vena cava (IVC) [12]. While the published series present only a short follow-up, no local or distant recurrences were observed. Based on these short-term data, no significant differences in long-term oncologic outcomes should be expected for RRN when compared to ON or LRN. However, a recent study [18], reported on the first case of tumor seeding in the omentum found in a female patient after previous transperitoneal RRN for papillary, low grade RCC (T2aN0M0). Two years after the robotic operation, the patient was diagnosed with cervical clear cell carcinoma and underwent radical hysterectomy with lymphadenectomy and omentectomy (with papillary RCC revealed in the pathological evaluation).

The rate of intra e postoperative complications was also low (Table 3). No perioperative deaths were reported. Postoperative pain evaluated by the 0–10 visual analogue scale (VAS) was generally low [8] (and mainly referred right after surgery). Use of analgesics was also low and limited to the day of surgery and the first postoperative day.

In the evaluated series, several potential advantages of the robotic approach on the pure laparoscopic one in the field of radical nephrectomy have been suggested:

1) The fourth robotic arm may provide upward retraction of the kidney, placing the renal hilum on stretch to facilitate a two-handed, precise dissection of the hilar vessels. Moreover, the correct use of the fourth robotic arm reduces the OT and minimizes the complexity of the laparoscopic tasks being performed by the bedside assistant to basic maneuvers such as suctioning, irrigating and changing of robotic instruments; thus, the console surgeon independence gets maximized.

2) The use of the articulated Hemolock applicator aids in the control of the kidney vessels under ideal angles, that sometimes results are reached with difficulty with a conventional laparoscopic Hemolock-clip applier.

3) The endowrist technology allows for an easy ligation of the kidney vessels, similar to ON.

4) Finally, some authors suggest the use of the robot in the field of RN as a training platform for more challenging surgical procedures, such as robotic partial nephrectomy [9].

However, the aforementioned advantages do not seem to provide better outcomes for the robotic approach over the traditional LRN/ON in the field of radical nephrectomy. In fact, the comparative studies between RRN and LRN [15-17] showed no differences in the evaluated outcomes except for a longer operative time (OT) for the robotic approach as evidenced in two studies [15,17].

It should be remembered, however, that the published series on RRN described the very first cases of the surgeons and consequently the outcomes may be influenced by the learning-curve issues.

Significantly higher costs were also observed for RRN: when the direct costs (i.e. instruments, nursing salaries, operative room and recovery room time) are evaluated, a total save of about 1300$ is observed for pure laparoscopy compared to RRN. Disposable instruments cost less in the pure laparoscopic group (1.573$ vs 1.942$), a difference that could be even higher if the surgeon spares the costs of the harmonic scalpel use (about 482 $ in this study) [16]. In a very recent study comparing costs between RRN and LRN [19], 24,312 radical nephrectomy cases performed either laparoscopically (68%) or robotically (32%) were analyzed. There was no demographic difference between RRN and LRN cases. Median total charges were $47,036 vs $38,068 for RRN vs LRN (p <0.001). Median total hospital costs for RRN were $15,149 compared to $11,735 for LRN (p <0.001). Compared to the laparoscopic approach robotic assistance conferred an estimated $4,565 and $11,267 increase in hospital costs and charges, respectively, when adjusted for adapted Charlson comorbidity index score, perioperative complications and length of stay (p <0.001). The authors concluded that RRN results in increased medical expense without improving patient morbidity.

The comparison between RRN and ON showed that ON is characterized by shorter OT, higher EBL, higher need of postoperative analgesics and longer hospital stay (HS) [15].

At the current state of the art and according to the most recent EAU guidelines on renal robotics [20], in ablative kidney surgery, robotics will produce no better outcomes compared to laparoscopy. The use of robotics in the field of RN for clinically localized RCC seems rather a technical overtreatment; thus, the use of pure laparoscopy to perform simple or radical nephrectomy is recommended (grade of recommendation C).

Anyway, there exist two potential areas of radical nephrectomy where the robotic approach may help to overcome the technical difficulties of the pure laparoscopy:

A) Field of R-LESS: the robotic platform provides the means to overcome technical constraints of the pure laparoscopic approach such as lack of triangulation, clashing of instruments and limited operating space. The comparison between R-LESS and conventional laparoscopy shows no difference in median OT, EBL, VAS and complication rates, while R-LESS may result in reduced inpatient narcotic requirements and HS compared to conventional LRN [14]. The promising results of this study should be interpreted with caution, because of issues related to the small number of the enrolled patients, to the retrospective design, to the short follow-up as well as to the absence of cases requiring adrenalectomy or significant hepatic or splenic retraction.

B) Field of RCC with associated IVC thrombi: Currently these cases are mainly performed through an open approach; some case-reports and small series on the use of pure laparoscopy have been published in this context, but in their majority the laparoscopic approach is used for the nephrectomy while the surgical time of thrombectomy is performed through an open incision [21,22] or with hand-assistance [23]. Some cases of pure or HA-laparoscopy for the entire procedure (i.e. nephrectomy and thrombectomy) have been described, limited to short thrombi [24,25].

In 2011 Abaza published the first series of five right radical nephrectomies plus thrombectomy for kidney tumors with IVC thrombus of various levels performed robotically [12]. “Simple” cases of short IVC thrombi as well as more “complex” cases requiring cross-clamping of the IVC and of the left renal vein were described. The results of the procedure were impressive, with a mean OT of 321 min and mean EBL of 170 ml for a mean tumor size of 10.4 cm. No transfusions and no complications were observed, while mean HS was only 1.2 days. The author, however, suggested that the procedure is challenging since serious and potentially fatal complications can occur. Extensive experience with robotic renal surgery and strong background in open urologic vascular surgery is suggested, while it should be recorded that the robotic surgeon is away from the bedside during such a critical procedure. To minimize the risk of tumor embolism, the renal artery should be preliminarily controlled preferably at the interaortocaval space in order to minimize the manipulation of the IVC. All the lumbar veins confluent to that segment of IVC should be clipped in order to guarantee a bloodless field. In cases of a short IVC thrombus, the fourth robotic arm may provide a lateral kidney retraction in order to pull the thrombus in the renal vein and allows for a subsequent application of a vascular stapler at the emergence of the renal vein, avoiding opening of the IVC.

Another case of RRN for a large renal mass with vena cava thrombus (cT3b), requiring a complete cross-clamping of the vena cava and entirely performed intracorporeally by relatively novice robotic surgeons was recently described [26].

Finally, it was recently demonstrated that robotic technology is associated with increased use of partial nephrectomy [27]; recent studies also document oncologic equivalence between partial and radical nephrectomies even for masses >7 cm [28,29]. Combining these data, an indirect increase in the number of RRN should be expected because of intraoperative conversions of procedures started with intent of nephron-sparing surgery (due to complications, technical difficulties or prolonged warm ischemia times), at least in the near future.

## Conclusions

Robotic nephrectomy is a feasible, safe and oncologically effective surgical treatment for clinically localized RCC. However, current literature does not provide any advantage for RRN if compared to standard laparoscopy; thus, RRN seems rather a “technical overtreatment”. Further future applications of robotics -in the field of radical nephrectomy- should be investigated where indications for open surgery still persist like the presence of an associated IVC thrombus.

## Competing interests

The authors declare that they have no competing interests.

## Authors’ contributions

ADA substantially contributed to: conception and design, acquisition of data, analysis and interpretation of data, drafting the manuscript and revising it critically for important intellectual content. RM substantially contributed to: analysis and interpretation of data, drafting of the manuscript. FA substantially contributed to: conception and design, revision for important intellectual content. SM substantially contributed to: conception and design, revision for important intellectual content. ES substantially contributed to: conception and design, revision for important intellectual content. BI substantially contributed to: conception and design, revision for important intellectual content. GV substantially contributed to: conception and design, revision for important intellectual content. RG substantially contributed to: analysis and interpretation of data, revision for important intellectual content. All authors read and approved the final manuscript.

## Pre-publication history

The pre-publication history for this paper can be accessed here:

http://www.biomedcentral.com/1471-2490/14/75/prepub
